# NLPR as a predictor of poor prognosis in patients with severe fever with thrombocytopenia syndrome: a prospective longitudinal study

**DOI:** 10.3389/fcimb.2026.1848735

**Published:** 2026-06-12

**Authors:** Chenxu Wang, Zhoujie Ma, Jilu Shen, Yan Zuo, Yuanhong Xu

**Affiliations:** 1 Department of Clinical Laboratory, The First Affiliated Hospital of Anhui Medical University, Hefei, Anhui, China; 2 Department of Clinical Laboratory, Anhui Public Health Clinical Center, Hefei, China

**Keywords:** albumin, mortality, NLPR, SFTS, systemic inflammatory indices

## Abstract

**Background and aims:**

Severe fever with thrombocytopenia syndrome (SFTS) is an emerging tick-borne infectious disease characterized by high mortality and rapid clinical progression. This longitudinal study aimed to investigate the temporal changes in laboratory parameters and evaluate the prognostic value of hematology-derived systemic inflammatory indices in patients with SFTS.

**Methods:**

Altogether, 235 hospitalized patients with SFTS were prospectively enrolled between 2022 and 2024. Demographic, clinical, treatment, and outcome data were collected and reassessed. Hematological, liver, and kidney function parameters, and myocardial parameters were routinely measured on days 0, 3, 7, and 10 after admission. Time-points stepwise Cox proportional hazards regression and receiver operating characteristic (ROC) curve analyses were performed at each time point to evaluate predictive performance.

**Results:**

Of the 235 patients, 61 (26.0%) died of SFTS. The median age was 64.0 (IQR 54.0-71.0) years, and 93 (39.6%) were male. After adjusting for confounding factors, serum creatinine (HR 1.017 [95% CI 1.006-1.029], *p* = 0.003), urea (HR 1.102 [95% CI 1.047-1.161], *p* < 0.001), platelet counts (HR 0.987 [95% CI 0.977-0.997], *p* = 0.010), neutrophil-to-lymphocyte-platelet ratio (NLPR) (HR 1.021 [95% CI 1.007-1.034], *p* = 0.002), and neutrophil percentage-to-albumin ratio (NPAR) (HR 1.689 [95% CI 1.028-2.776], *p* = 0.039) were identified as independently associated with prognosis at each time point. Among them, NLPR presented consistently high predictive performance, with an area under the curve (AUC) of 0.802 (95% CI 0.736-0.868) on day 0, 0.833 (95% CI 0.774-0.892) on day 3, 0.861 (95% CI 0.784-0.938) on day 7, and 0.840 (95% CI 0.706-0.975) on day 10. Patients with high NLPR (> 3.76) had significantly shorter survival times (HR 6.584 [95% CI 3.840-11.290], *p* < 0.001 by the log-rank test). In addition, albumin treatment was associated with a reduced risk of mortality in the high-NLPR group (HR 0.423 [95% CI 0.195-0.919], *p* = 0.030).

**Conclusions:**

NLPR may serve as a simple and effective prognostic marker for SFTS. Albumin treatment may be associated with a lower risk of mortality in patients with a high NLPR.

## 1 Introduction

Severe fever with thrombocytopenia syndrome (SFTS) was first identified in Hubei and Henan Provinces, China, in 2009 and has subsequently been reported in Japan, South Korea, and other countries (Liu et al., 2014). SFTS is an emerging tick-borne infectious disease caused by Dabie bandavirus (DBV) (Sasaya et al., 2023). Clinically, the virus causes various symptoms, such as fever, thrombocytopenia, leukopenia, gastrointestinal symptoms, and multiple organ failure (Li et al., 2021). A systematic review reported a pooled case-fatality rate of 18% for SFTS (He et al., 2020). Unfortunately, there is currently no specific antiviral therapy or licensed vaccine available for SFTS (Kim et al., 2024; Charoensakulchai et al., 2025). Therefore, early identification of high-risk patients is essential for improving clinical outcomes.

Advanced age, male sex, and coinfection have been reported to be associated with fatal outcomes in SFTS (Zhao et al., 2021). Previous prognostic studies based on laboratory parameters identified several effective predictors of poor outcomes in patients with SFTS, including lactate dehydrogenase, aspartate aminotransferase, and D-dimer (Meng et al., 2024; Xia et al., 2024). In addition, scoring systems have been developed to predict the severity of SFTS (Chen et al., 2025). Nevertheless, SFTS progresses rapidly during the acute phase, and Wang et al. (2021) reported that laboratory parameters change dynamically during its course and are closely associated with transcriptional regulation. However, most previous studies were cross-sectional and relied primarily on admission data, which may have limited their clinical utility. Therefore, we conducted a longitudinal study at key clinical evaluation time points after hospital admission to identify effective predictors that remained informative throughout the disease course.

SFTS is characterized by a systemic inflammatory response syndrome (SIRS) associated with viral replication (Zhang et al., 2024). Systemic inflammatory indices have been increasingly recognized as useful tools for risk stratification of various inflammatory and infectious diseases (Briciu et al., 2025). However, few studies have investigated the value of systemic inflammatory indices in predicting the clinical outcomes of SFTS, with inconsistent findings (Liu et al., 2022; Yang et al., 2025). The neutrophil-to-lymphocyte-platelet ratio (NLPR) has recently demonstrated promising diagnostic and prognostic value in infectious diseases (Shi et al., 2022; Umut et al., 2023). The NLPR reflects multiple pathophysiological processes, including innate immune activation, adaptive immune suppression, and platelet-related coagulation disturbances (Wang et al., 2024). Nevertheless, evidence regarding the predictive value of systemic inflammatory indices in SFTS remains limited, especially with respect to stage-based prognostic assessment. Considering the rapid progression of SFTS, evaluation of these indices at different clinical stages may offer more clinically prognostic information than assessment at admission alone.

In this prospective longitudinal observational study, we assessed the association between the dynamic laboratory parameters and clinical outcomes in patients with SFTS, particularly on the prognostic value of systemic inflammatory indices for mortality. This study may provide a simple and practical approach for early identification of high-risk patients and support risk-stratified management of SFTS.

## 2 Materials and methods

### 2.1 Study design and participants

Overall, 235 patients diagnosed with SFTS at the First Affiliated Hospital of Anhui Medical University, China, between May 2022 and September 2024 were prospectively enrolled. SFTS was diagnosed based on at least one of the following criteria: (1) positive DBV nucleic acid detected by RT-PCR, (2) isolation of DBV from clinical specimens, (3) seroconversion of DBV-specific IgG or a ≥ 4-fold increase in antibody titer in the convalescent phase compared with the acute phase. All diagnostic tests for DBV were performed in department of clinical laboratory in our hospital. DBV RNA was detected by real-time RT−PCR using a DBV nucleic acid detection kit (Beijing BGI-GBI Biotech Co., Ltd., Beijing, China) on the Applied Biosystems 7500 Real-Time PCR System (Thermo Fisher Scientific, Waltham, MA, USA). Serological testing for DBV−specific antibodies was performed using a DBV IgM/IgG antibody detection kit based on the immune colloidal gold technique (Zhongshan Bioengineering Co., Ltd., Zhongshan, China). These assays were all commercially approved for clinical diagnostic use. The exclusion criteria were as follows: (1) patients discharged within 48 h of admission, (2) patients with incomplete clinical data, (3) patients with malignant tumors, and (4) patients aged < 18 years.

**Figure 1:**
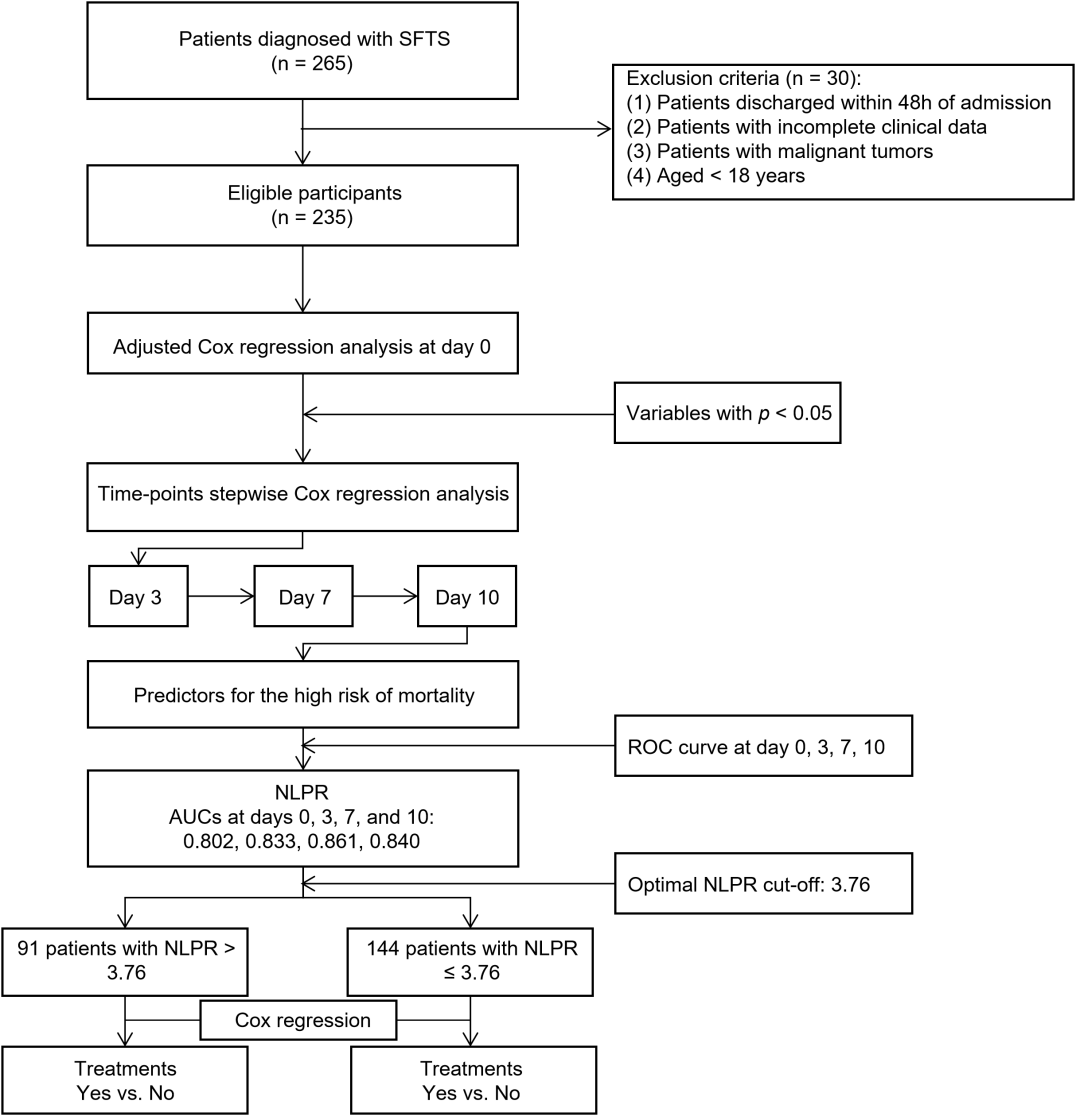
Study flowchart of the present study. SFTS, severe fever with thrombocytopenia syndrome; NLPR, neutrophil-to-lymphocyte-platelet ratio.

This study was conducted in accordance with the Declaration of Helsinki and was approved by the First Affiliated Hospital of Anhui Medical University. Written informed consent to participate in this study was provided by the participants’ legal guardians or their next of kin. A flowchart of the study is shown in **Figure 1**.

### 2.2 Data collection

According to Chinese management guidelines for SFTS and previous studies on SFTS, in this longitudinal study, we determined four clinical time points (days 0, 3, 7, and 10) after systematically assessing the different clinical stages, time from onset to admission, the clinician’s assessment of the disease status, and samples accessibility (Chinese Center for Disease Control and Prevention, 2024) (Hao et al., 2025). Meanwhile, to fully reflect the changes throughout the disease course and reduce the impact of missing samples, we set a time window of ±1 day for each time point. Epidemiological, demographic, clinical, treatment, and outcome data were obtained from electronic medical records and reassessed. After admission, hematological parameters, coagulation indices, liver and kidney function markers, and myocardial biomarkers were routinely measured. Hematological parameters were measured using the Sysmex XN-9000 automated hematology system (Sysmex Corporation, Kobe, Japan). Biochemical parameters, including liver and kidney function markers and myocardial markers, were measured using the Roche Cobas 8000 automated biochemical system (Roche Diagnostics, Mannheim, Germany). The laboratory was certified according to the ISO 15189 standard. The endpoint of the study was defined as death or discharge. Patients were followed up for 28 days after admission through in-hospital assessment and post-discharge follow-up. The systemic inflammatory indices analyzed in this study are summarized in **Table 1**.

**Table 1:** Full names, abbreviations, and formulas of systemic inflammatory indices.

Full name	Abbreviation	Formula
Neutrophil-to-lymphocyte ratio	NLR	neutrophil count/lymphocyte count
Platelet-to-lymphocyte ratio	PLR	platelet count/lymphocyte count
Lymphocyte-to-monocyte ratio	LMR	lymphocyte count/monocyte count
Platelet-to-neutrophil ratio	PNR	platelet count/neutrophil count
Platelet-to-monocyte ratio	PMR	platelet count/monocyte count
Neutrophil-to-lymphocyte-platelet ratio	NLPR	neutrophil count × 100/ (lymphocyte count × platelet count)
Neutrophil percentage-to-albumin ratio	NPAR	neutrophil percentage/albumin
Systemic immune-inflammation index	SII	platelet count × neutrophil count/lymphocyte count
Systemic inflammation response index	SIRI	neutrophil count × monocyte count/lymphocyte count
Aggregate index of systemic inflammation	AISI	neutrophil count × monocyte count × platelet count/lymphocyte count

Neutrophil count, lymphocyte count, monocyte count, and platelet count are expressed as ×10^9^/L; albumin is expressed as g/L; neutrophil percentage is expressed as %.

### 2.3 Statistical analysis

All statistical analyses were performed using SPSS version 27.0 (SPSS Inc., Chicago, IL, USA) and GraphPad Prism version 10.0 (GraphPad Software Inc., La Jolla, CA). Continuous variables were tested for normality using the Kolmogorov-Smirnov test and expressed as mean ± standard deviation (SD) or median (interquartile range [IQR]), as appropriate. Differences between groups were compared using the independent samples *t*-test or Mann-Whitney U test for continuous variables and the chi-squared test or Fisher’s exact test for categorical variables.

To evaluate which indicators retained prognostic value in patients with SFTS and maintained predictive capability throughout the disease course, a time-points stepwise Cox regression model was used in this longitudinal study. Briefly, variables with *p* values < 0.05 in comparison analysis between the two groups were included in the Cox regression analysis on day 0. After adjusting for age, sex, history of drinking, history of smoking, cardiovascular and cerebrovascular diseases, and diabetes, variables with *p* values < 0.05 in the adjusted multivariate Cox regression analysis on day 0 were then included in the multivariate Cox regression analysis on day 3. Similarly, variables with *p* values < 0.05 in adjusted multivariate Cox regression analysis at a former time point were included in the analysis on the next time point until day 10. Hazard ratios (HRs) and corresponding 95% confidence intervals (CIs) were reported as effect measures for Cox regression analysis. The proportional hazards assumption was tested using Schoenfeld residuals before Cox regression analysis. Survival time was defined as the interval between admission and the outcome event. Receiver operating characteristic (ROC) curve analysis was performed to determine the optimal cutoffs for each risk factor, with the maximum Youden index (sensitivity + specificity - 1) used as the primary criterion. The area under the curve (AUC) was calculated to assess predictive performance. Kaplan-Meier survival analysis was performed to estimate the cumulative risk of death, and differences between the groups were compared using the log-rank test. The relationship between NLPR and other clinical parameters was determined through Spearman rank correlation analysis.

To avoid a potential reduction in statistical power and bias associated with the direct exclusion of missing values, this study employed multiple imputation using chained equations for missing data estimation during the initial hospitalization. This method iteratively generates complete datasets by constructing a multivariate statistical model, ultimately producing five imputed complete datasets, on which all subsequent statistical analyses are based. A *p*-value (two-sided) of less than 0.05 was considered statistically significant.

## 3 Results

### 3.1 Demographic and clinical characteristics of patients with SFTS

After excluding patients who did not meet the inclusion criteria, 235 patients were enrolled in this study, of whom 61 (26.0%) died. The demographic and baseline characteristics of the patients are summarized in **Table 2**. Patients’ ages ranged from 23.0 to 85.0 years, with a median age of 64.0 (IQR, 54.0-71.0) years. Altogether, 93 patients (39.6%) were male. Compared with survivors, non-survivors were older (*p* < 0.001) and had a significantly higher proportion of cardiovascular diseases (*p* < 0.001). Regarding treatment, 175 (74.5%) patients received intravenous immunoglobulin, and 157 (66.8%) patients received platelet transfusion. The non-specific symptoms are shown in **Supplementary Table 1**. Furthermore, non-survivors had a significantly shorter median length of hospital stay than survivors (6.0 [IQR 4.0-11.0] vs. 11.0 [IQR 9.0-15.0] days, *p* < 0.001). Fatal cases had higher intensive care unit (ICU) admission rates (*p* < 0.001).

**Table 2:** Demography, clinical, and laboratory findings between survivors and non-survivors with SFTS.

Variables	Total (n = 235)	Survivors (n = 174)	Non-survivors (n = 61)	*P* value
Demography				
Age (years)	64.0 (54.0-71.0)	61.0 (53.0-69.0)	69.0 (62.0-75.0)	**<0.001**
Gender, n (%)	93 (39.6)	66 (37.9)	27 (44.3)	0.384
Bite history, n (%)	80 (34.0)	59 (33.9)	21 (34.4)	0.941
Contact history, n (%)	7 (3.0)	7 (4.0)	0 (0.0)	0.195
Smoking history, n (%)	21 (8.9)	14 (8.0)	7 (11.5)	0.419
Drinking history, n (%)	20 (8.5)	14 (8.0)	6 (9.8)	0.666
Baseline diseases, n (%)				
Cardiovascular diseases	50 (21.3)	29 (16.7)	21 (34.4)	**0.004**
Diabetes	27 (11.50)	19 (10.9)	8 (13.1)	0.644
Cerebral infarction	31 (13.2)	19 (10.9)	12 (19.7)	0.129
Liver diseases	8 (3.4)	5 (2.9)	3 (4.9)	0.431
Kidney diseases	15 (6.4)	9 (5.2)	6 (9.8)	0.328
Laboratory parameters				
Serum creatinine (μmol/L)	72.0 (59.3-91.5)	67.6 (57.7-83.5)	81.0 (66.5-111.6)	**<0.001**
Urea (mmol/L)	5.7(4.5-7.7)	5.0 (4.3-6.8)	7.7 (6.2-10.0)	**<0.001**
Uric acid (μmol/L)	273.5 ± 8.0	259.8 ± 107.1	312.5 ± 151.5	**0.004**
Alanine aminotransferase (U/L)	66.0 (38.0-112.0)	64.0 (36.0-95.5)	81.0 (41.9-147.0)	**0.026**
Aspartate transaminase (U/L)	133.0 (66.0-250.0)	115.5 (60.8-210.5)	229.0 (103.0-562.9)	**<0.001**
GGT (U/L)	32.0 (19.0-67.0)	32.0 (19.0-57.3)	34.0 (19.0-76.5)	0.329
Alkaline phosphatase (U/L)	69.0 (55.0-85.0)	69.0 (56.0-83.3)	66.0 (53.5-92.0)	0.661
DBIL (μmol/L)	3.9 (2.8-5.9)	3.9 (2.8-5.6)	4.5 (2.8-7.6)	0.218
IBIL (μmol/L)	5.8 (4.7-7.7)	5.8 (4.7-7.9)	5.8 (4.6-7.1)	0.313
Serum albumin (g/L)	36.0 (32.0-39.9)	37.0 (32.7-40.6)	34.2 (30.9-37.1)	**<0.001**
Serum globulin (g/L)	28.6 ± 0.3	28.8 ± 5.2	28.1 ± 4.2	0.378
Platelet (× 10^9^/L)	58.0 (38.0-87.0)	62.0 (43.0-95.3)	41.0 (30.5-63.5)	**<0.001**
White blood cell (× 10^9^/L)	2.4 (1.7-3.7)	2.3 (1.7-3.9)	2.5 (1.6-3.5)	0.808
Neutrophil (× 10^9^/L)	1.3 (0.8-2.3)	1.2 (0.8-2.3)	1.6 (0.9-2.6)	0.146
Lymphocyte (× 10^9^/L)	0.7 (0.5-1.1)	0.8 (0.6-1.2)	0.5 (0.4-0.7)	**<0.001**
Monocyte (× 10^9^/L)	0.1 (0.1-0.3)	0.2 (0.1-0.3)	0.1 (0.1-0.2)	**<0.001**
Creatine kinase (U/L)	298.0 (125.0-847.0)	259.0 (113.5-786.0)	480.0 (194.0-1272.0)	**0.009**
CK-MB (U/L)	14.0 (6.0-29.0)	13.0 (6.0-26.3)	18.0 (6.0-43.5)	0.236
Lactate dehydrogenase (U/L)	650.0 (388.0-1297.0)	579.5 (364.5-1080.3)	1147.0 (565.0-2505.0)	**<0.001**
Treatments				
Ribavirin	122 (51.9)	87 (50.0)	35 (57.4)	0.321
Albumin	52 (26.2)	36 (20.7)	16 (26.2)	0.370
Platelet	157 (66.8)	110 (74.0)	47 (77.0)	**0.048**
Intravenous immunoglobulin	175 (74.5)	119 (68.4)	56 (91.8)	**<0.001**
Corticosteroids	34 (14.5)	24 (13.8)	10 (16.4)	0.619
Outcomes				
Duration of Hospital Stay (days)	11.0 (7.0-14.0)	11.0 (9.0-15.0)	6.0 (4.0-11.0)	**<0.001**
Admitted to ICU, n (%)	17 (7.2)	3 (1.7)	14 (23.0)	**<0.001**

Data were expressed as mean ± standard deviation or median (IQR) or n (%) and were compared using the Chi-square test, Mann-Whitney U test, or Student’s *t*-test as appropriate.

IQR, interquartile range and Bold values indicate statistical significance; SFTS, severe fever with thrombocytopenia syndrome; GGT, γ-glutamyl transferase; DBIL, direct bilirubin; IBIL, indirect bilirubin; CK-MB, creatine kinase isoenzyme.

### 3.2 Comparisons of laboratory findings between survivors and non-survivors with SFTS

Next, we compared laboratory findings on the day of admission between survivors and non-survivors with SFTS. As shown in **Table 2**, non-survivors showed more pronounced hematological abnormalities, as reflected by lower platelet [41.0 (30.5-63.5) vs. 62.0 (43.0-95.3) ×10^9^/L, *p* < 0.001], lymphocyte [0.5 (0.4-0.7) vs. 0.8 (0.6-1.2) ×10^9^/L, *p* < 0.001], and monocyte [0.1 (0.1-0.2) vs. 0.2 (0.1-0.3) ×10^9^/L, *p* < 0.001] counts than survivors. Renal function markers, including serum creatinine (Scr) [81.0 (66.5-111.6) vs. 67.6 (57.7-83.5) μmol/L, *p* < 0.001], urea [7.7 (6.2-10.0) vs. 5.0 (4.3-6.8) mmol/L, *p* < 0.001], and uric acid [312.5 ± 151.5 vs. 259.8 ± 107.1 μmol/L, *p* = 0.004], were significantly higher in non-survivors. In addition, non-survivors had higher levels of markers related to liver injury and tissue damage, including alanine aminotransferase [81.0 (41.9-147.0) vs. 64.0 (36.0-95.5) U/L, *p* = 0.026], aspartate aminotransferase [229.0 (103.0-562.9) vs. 115.5 (60.8-210.5) U/L, *p* < 0.001], creatine kinase [480.0 (194.0-1272.0) vs. 259.0 (113.5-786.0) U/L, *p* = 0.009], and lactate dehydrogenase [1147.0 (565.0-2505.0) vs. 579.5 (364.5-1080.3) U/L, *p* < 0.001]. Serum albumin levels [34.2 (30.9-37.1) vs. 37.0 (32.7-40.6) g/L, *p* < 0.001] were also significantly lower in non-survivors.

### 3.3 Systemic inflammatory indices of patients with SFTS

Systemic inflammatory indices previously reported as predictors of mortality in infectious diseases were evaluated in this study (Briciu et al., 2025). The results are shown in **Table 3**. We further compared the systemic inflammatory indices between survivors and non-survivors. As shown in **Table 3**, compared with survivors, non-survivors had significantly higher NLR [2.9 (1.8-4.0) vs. 1.6 (1.0-2.5), *p* < 0.001], NLPR [6.5 (3.7-15.1) vs. 2.7 (1.61-4.03), *p* < 0.001], NPAR [2.0 ± 0.6 vs. 1.5 ± 0.5, *p* < 0.001], and lower platelet-to-neutrophil ratio (PNR) [31.4 (11.8-54.4) vs. 51.0 (30.2-82.9), *p* < 0.001] at admission.

**Table 3:** Systemic inflammatory indices between the survivors and non-survivors with SFTS.

Indices	Total (n = 235)	Survivors (n = 174)	Non-survivors (n = 61)	*P* value
NLR	1.8 (1.1-3.0)	1.6 (1.0-2.5)	2.9 (1.8-4.0)	**<0.001**
PLR	83.1 (46.3-135.9)	80.8 (46.2-137.3)	89.5 (48.6-134.9)	0.830
LMR	4.9 (3.2-7.7)	4.9 (3.2-7.7)	5.1 (3.6-7.7)	0.716
PNR	45.3 (25.7-76.3)	51.0 (30.2-82.9)	31.4 (11.8-54.4)	**<0.001**
PMR	430.0 (235.0-750.0)	423.0 (232.5-719.7)	450.0 (256.8-870.0)	0.418
NLPR	3.1 (1.9-5.0)	2.7 (1.61-4.03)	6.5 (3.7-15.1)	**<0.001**
NPAR	1.7 ± 0.1	1.5 ± 0.5	2.0 ± 0.6	**<0.001**
SII	108.6 (58.2-215.1)	107.8 (56.3-207.2)	120.8 (66.8-235.6)	0.331
SIRI	0.3 (0.1-0.6)	0.3 (0.1-0.5)	0.3 (0.1-0.6)	0.646
AISI	15.6 (6.3-38.4)	17.0 (7.0-41.3)	11.2 (5.1-26.5)	0.057

Data were expressed as means ± standard deviation or median (IQR). Bold values indicate statistical significance. NLR, neutrophil-to-lymphocyte ratio; PLR, platelet-to-lymphocyte ratio; LMR, lymphocyte-to-monocyte ratio; PNR, platelet-to-neutrophil Ratio; PMR, platelet-to-monocyte ratio; NLPR, neutrophil-to-lymphocyte-platelet ratio; NPAR, neutrophil percentage-to-albumin ratio; SII, systemic immuneinflammation index; SIRI, systemic inflammatory response index; AISI, aggregate index of systemic inflammation.

### 3.4 Temporal changes of laboratory findings and systemic inflammatory indices of patients with SFTS during hospitalization

**Figure 2:**
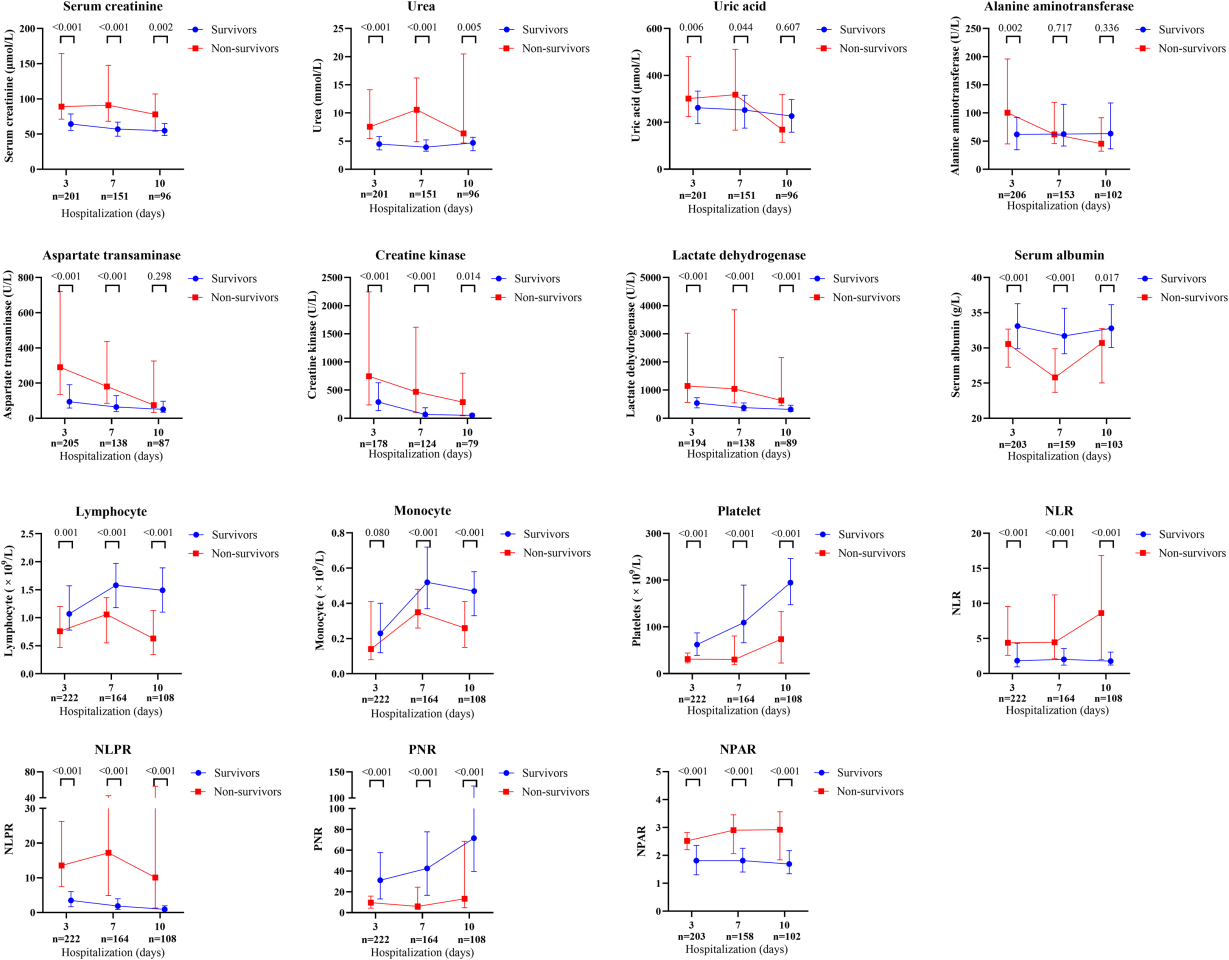
Temporal changes of laboratory parameters and systemic inflammatory indices in survivors and non-survivors with SFTS during hospitalization. Data were presented as median (interquartile range). The *p* values are shown at the top of each graph. n means sample size. SFTS, severe fever with thrombocytopenia syndrome; NLR, neutrophil-to-lymphocyte ratio; NLPR, neutrophil-to-lymphocyte-platelet ratio; PNR, platelet-to-neutrophil ratio; NPAR, neutrophil percentage-to-albumin ratio.

To clarify the dynamic patterns during SFTS progression, parameters with *p* < 0.05 in **Table 2** and **3** were then evaluated at days 3, 7, and 10 after admission. Temporal changes, statistical comparisons, and sample sizes at each time point are shown in **Figure 2**. Non-survivors showed a similar trend in renal function, with the most pronounced between-group differences observed on day 7. Liver and myocardial enzyme profiles gradually decreased during hospitalization, and the differences between the two groups gradually narrowed. Interestingly, the differences in platelet and lymphocyte counts and NLR between the two groups peaked on day 10. Notably, the NLPR remained significantly higher in non-survivors throughout the observation period, with the greatest difference observed on day 7.

### 3.5 Time-points stepwise Cox regression analysis for prognosis in patients with SFTS

Variables with *p* values < 0.05 in **Table 2** and **3** were then included in a time-points Cox regression model. As shown in **Table 4**, after adjusting for confounding factors in model 2 (age, sex, history of drinking, history of smoking, cardiovascular and cerebrovascular diseases, and diabetes), multiple factors were associated with prognosis on day 0, including higher levels of Scr, urea, uric acid, alanine aminotransferase, aspartate aminotransferase, creatine kinase, lactate dehydrogenase, monocyte counts, NLR, NLPR, and NPAR, as well as decreased levels of lymphocyte and platelet counts, and PNR. As described above, variables with *p* values < 0.05 in the adjusted Cox regression analysis on a former time point was then included in an adjusted multivariate Cox regression analysis on a next time point. Ultimately, after adjusting for model 2 on day 10, we identified that Scr (adjusted HR 1.017 [95% CI 1.006-1.029], *p* = 0.003), urea (adjusted HR 1.102 [95% CI 1.047-1.161], *p* < 0.001), platelets (adjusted HR 0.987 [95% CI 0.977-0.997], *p* = 0.010), NPAR (adjusted HR 1.689 [95% CI 1.028-2.776], *p* = 0.039), and NLPR (adjusted HR 1.021 [95% CI 1.007-1.034], *p* = 0.002) remained independently associated with poor prognosis throughout the evaluated time points in patients with SFTS.

**Table 4:** Time-points stepwise Cox regression analysis for prognosis in patients with SFTS.

Variables	HR (95%CI)	*P* value	Adjusted HR (95%CI) ^model1^	*P* value	Adjusted HR (95%CI) ^model2^	*P* value
Day 0 (n = 235)						
Age	1.055 (1.026-1.085)	<0.001				
Sex	1.212 (0.726-2.023)	0.462				
Scr	1.007 (1.003-1.010)	<0.001	1.006(1.002-1.010)	0.002	1.007(1.003-1.011)	**0.001**
Urea	1.105 (1.059-1.153)	<0.001	1.088(1.036-1.142)	<0.001	1.106(1.047-1.167)	**<0.001**
Urea acid	1.002 (1.001-1.004)	0.004	1.002(1.001-1.004)	0.007	1.003(1.001-1.005)	**0.006**
ALT	1.002 (1.001-1.004)	<0.001	1.003(1.002-1.005)	<0.001	1.003 (1.002-1.005)	**<0.001**
AST	1.001 (1.000-1.001)	<0.001	1.001(1.000-1.001)	<0.001	1.001 (1.000-1.001)	**<0.001**
ALB	0.943 (0.901-0.987)	0.012	0.951 (0.905-0.999)	0.045	0.950 (0.902-1.000)	0.050
Platelets	0.982 (0.972-0.992)	<0.001	0.984 (0.975-0.994)	0.001	0.984 (0.975-0.994)	**0.001**
Lymphocyte	0.190 (0.085-0.423)	<0.001	0.204 (0.089-0.464)	<0.001	0.209 (0.091-0.479)	**<0.001**
Monocyte	0.048 (0.006-0.394)	0.005	0.043 (0.005-0.400)	0.006	0.036 (0.004-0.352)	**0.004**
CK	1.000 (1.000-1.000)	0.017	1.000 (1.000-1.000)	0.002	1.000 (1.000-1.000)	**0.006**
LDH	1.000 (1.000-1.000)	<0.001	1.000 (1.000-1.000)	<0.001	1.000 (1.000-1.000)	**<0.001**
NLR	1.176 (1.105-1.252)	<0.001	1.154 (1.086-1.226)	<0.001	1.158 (1.089-1.232)	**<0.001**
PNR	0.986 (0.977-0.994)	0.001	0.985 (0.976-0.994)	<0.001	0.984 (0.975-0.993)	**<0.001**
NLPR	1.004 (1.030-1.058)	<0.001	1.041 (1.027-1.056)	<0.001	1.049 (1.031-1.067)	**<0.001**
NPAR	2.677 (1.733-4.136)	<0.001	2.369 (1.547-3.627)	<0.001	2.496 (1.606-3.880)	**<0.001**
Day 3						
Age (222)	1.054 (1.024-1.084)	<0.001				
Sex (222)	1.156 (0.686-1.946)	0.586				
Scr (201)	1.009 (1.006-1.012)	<0.001	1.011 (1.008-1.014)	<0.001	1.012 (1.008-1.015)	**<0.001**
Urea (201)	1.134 (1.092-1.178)	<0.001	1.133 (1.088-1.181)	<0.001	1.161 (1.103-1.222)	**<0.001**
Urea acid (201)	1.003 (1.001-1.004)	<0.001	1.003 (1.001-1.004)	<0.001	1.003 (1.002-1.005)	**<0.001**
ALT (206)	1.003 (1.001-1.005)	<0.001	1.005 (1.003-1.007)	<0.001	1.005 (1.003-1.007)	**<0.001**
AST (205)	1.001 (1.000-1.001)	<0.001	1.001 (1.001-1.002)	<0.001	1.001 (1.001-1.002)	**<0.001**
Platelets (222)	0.970 (0.957-0.983)	<0.001	0.972 (0.960-0.985)	<0.001	0.973 (0.959-0.986)	**<0.001**
Lymphocyte (222)	1.074 (0.921-1.252)	0.363	1.072 (0.953-1.206)	0.249	1.043 (0.908-1.198)	0.554
Monocyte (222)	1.072 (0.417-2.761)	0.885	1.210 (0.456-3.210)	0.702	1.275 (0.457-3.556)	0.643
CK (178)	1.000 (1.000-1.000)	<0.001	1.000 (1.000-1.000)	<0.001	1.000 (1.000-1.000)	**<0.001**
LDH (194)	1.000 (1.000-1.000)	0.004	1.000 (1.000-1.000)	<0.001	1.000 (1.000-1.000)	**<0.001**
NLR (222)	1.067 (1.028-1.108)	<0.001	1.057 (1.017-1.098)	0.004	1.064 (1.018-1.111)	**0.005**
PNR (222)	0.958 (0.938-0.977)	<0.001	0.961 (0.942-0.980)	<0.001	0.958 (0.939-0.978)	**<0.001**
NLPR (222)	1.019 (1.013-1.025)	<0.001	1.016 (1.010-1.023)	<0.001	1.017 (1.010-1.024)	**<0.001**
NPAR (203)	2.061 (1.585-2.682)	<0.001	2.195 (1.603-3.004)	<0.001	2.166 (1.602-2.928)	**<0.001**
Day 7						
Age (164)	1.038 (0.997-1.080)	0.069				
Sex (164)	0.665 (0.302-1.465)	0.311				
Scr (151)	1.007 (1.004-1.009)	<0.001	1.008 (1.005-1.011)	<0.001	1.009 (1.005-1.021)	**<0.001**
Urea (151)	1.091 (1.057-1.126)	<0.001	1.088 (1.054-1.123)	<0.001	1.097 (1.061-1.135)	**<0.001**
Urea acid (151)	1.004 (1.002-1.006)	<0.001	1.004 (1.002-1.006)	<0.001	1.004 (1.002-1.006)	**<0.001**
ALT (153)	1.001 (1.001-1.004)	<0.001	1.002 (1.001-1.004)	<0.001	1.002 (1.001-1.004)	**0.003**
AST (138)	1.001 (1.000-1.001)	<0.001	1.001 (1.000-1.001)	<0.001	1.001 (1.000-1.001)	**0.002**
Platelets (164)	0.980 (0.969-0.991)	<0.001	0.981 (0.970-0.991)	<0.001	0.980 (0.969-0.991)	**<0.001**
CK (124)	1.001 (1.000-1.001)	<0.001	1.001 (1.000-1.001)	<0.001	1.001 (1.001-1.002)	**<0.001**
LDH (138)	1.000 (1.000-1.000)	0.152	1.000 (1.000-1.000)	0.193	1.000 (1.000-1.000)	0.101
NLR (164)	1.020 (0.998-1.043)	0.070	1.018 (0.993-1.043)	0.159	1.017 (0.991-1.043)	0.199
PNR (164)	0.967 (0.946-0.988)	0.002	0.969 (0.948-0.991)	0.005	0.969 (0.947-0.991)	**0.007**
NLPR (164)	1.016 (1.009-1.024)	<0.001	1.015 (1.007-1.024)	<0.001	1.017 (1.008-1.027)	**<0.001**
NPAR (158)	1.699 (1.359-2.125)	<0.001	1.643 (1.289-2.093)	<0.001	1.634 (1.243-2.148)	**<0.001**
Day 10						
Age (107)	0.991 (0.944-1.041)	0.730				
Sex (107)	0.837 (0.253-2.764)	0.770				
Scr (95)	1.013 (1.006-1.021)	<0.001	1.015 (1.007-1.023)	<0.001	1.017 (1.006-1.029)	**0.003**
Urea (95)	1.097 (1.051-1.146)	<0.001	1.096 (1.050-1.145)	<0.001	1.102 (1.047-1.161)	**<0.001**
Urea acid (95)	1.003 (0.998-1.007)	0.214	1.003 (0.998-1.007)	0.254	1.003 (0.998-1.007)	0.206
ALT (101)	0.996 (0.984-1.008)	0.475	0.993 (0.980-1.007)	0.317	0.995 (0.982-1.008)	0.430
AST (73)	1.004 (1.001-1.007)	0.014	1.004 (1.000-1.007)	0.038	1.003 (0.999-1.007)	0.105
Platelets (107)	0.990 (0.982-0.997)	0.007	0.990 (0.982-0.997)	0.008	0.987 (0.977-0.997)	**0.010**
CK (68)	1.000 (1.000-1.001)	0.517	1.000 (1.000-1.001)	0.623	1.000 (1.000-1.001)	0.546
PNR (107)	0.987 (0.972-1.002)	0.096	0.987 (0.971-1.002)	0.088	0.986 (0.971-1.002)	0.077
NLPR (107)	1.018 (1.009-1.026)	<0.001	1.018 (1.009-1.027)	<0.001	1.021 (1.007-1.034)	**0.002**
NPAR (101)	1.847 (1.176-2.901)	0.008	1.833 (1.191-2.819)	0.006	1.689 (1.028-2.776)	**0.039**

Model 1: adjusted by age and sex.

Model 2: adjusted by age, sex, history of drinking, history of smoking, cardiovascular and cerebrovascular diseases, and diabetes.

Variables (n), n: available cases; CI, confidence interval; Bold values indicate statistical significance; Scr, serum creatinine; ALT, alanine aminotransferase; AST, aspartate aminotransferase; ALB, albumin; CK, creatine kinase; LDH, lactate dehydrogenase. NLR, neutrophil-to-lymphocyte ratio; PNR, platelet-to-neutrophil Ratio; NLPR, neutrophil-to-lymphocyte-platelet ratio; NPAR, neutrophil percentage-to-albumin ratio.

### 3.6 NLPR was identified as a predictor for the prognosis of patients with SFTS

**Figure 3:**
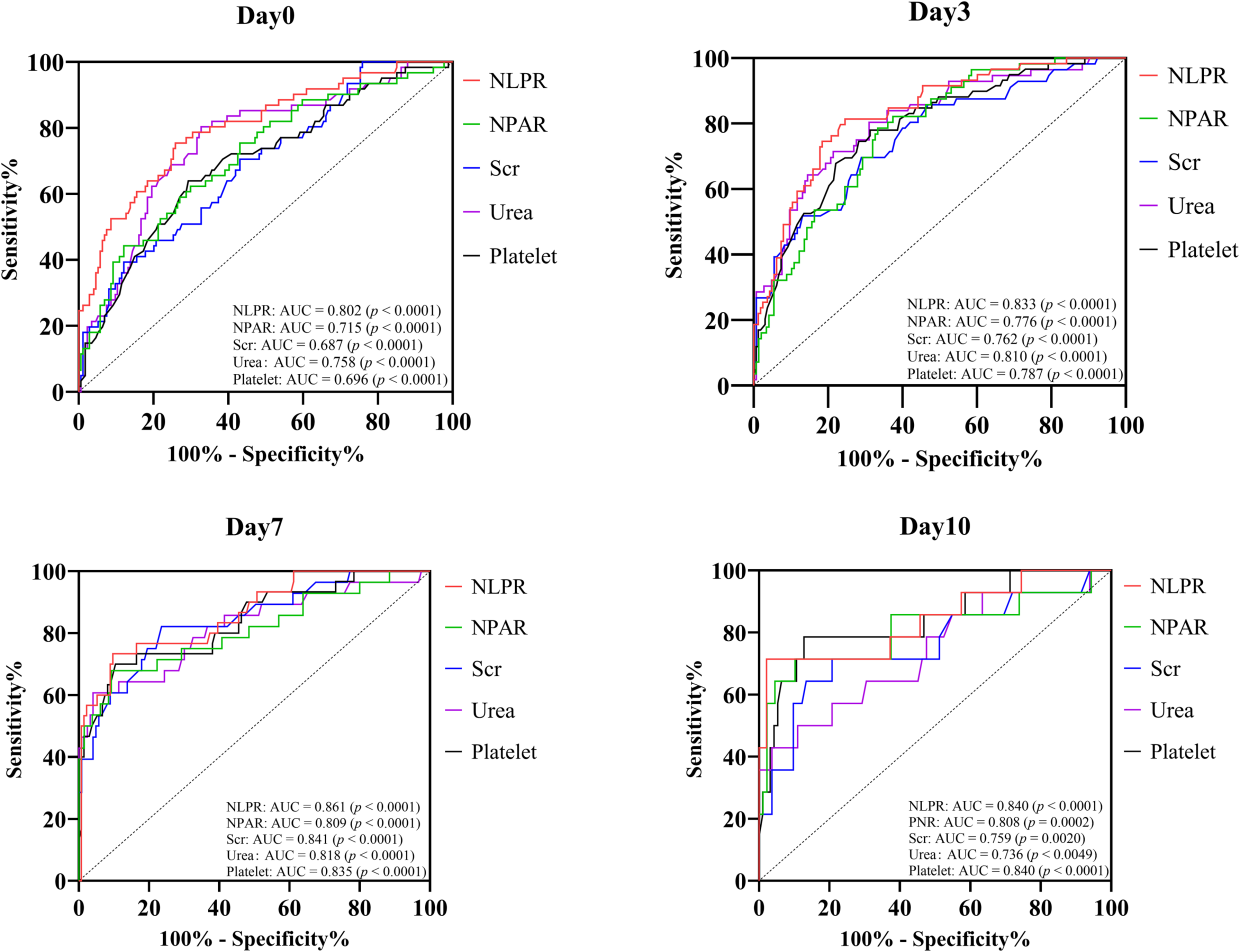
Receiver operating characteristic (ROC) curves for evaluating the predictive ability for 28-day mortality of SFTS at different time points. SFTS, severe fever with thrombocytopenia syndrome; AUC, area under the ROC curve; NLPR, neutrophil-to-lymphocyte-platelet ratio; NPAR, neutrophil percentage-to-albumin ratio; Scr, Serum creatinine.

**Figure 4:**
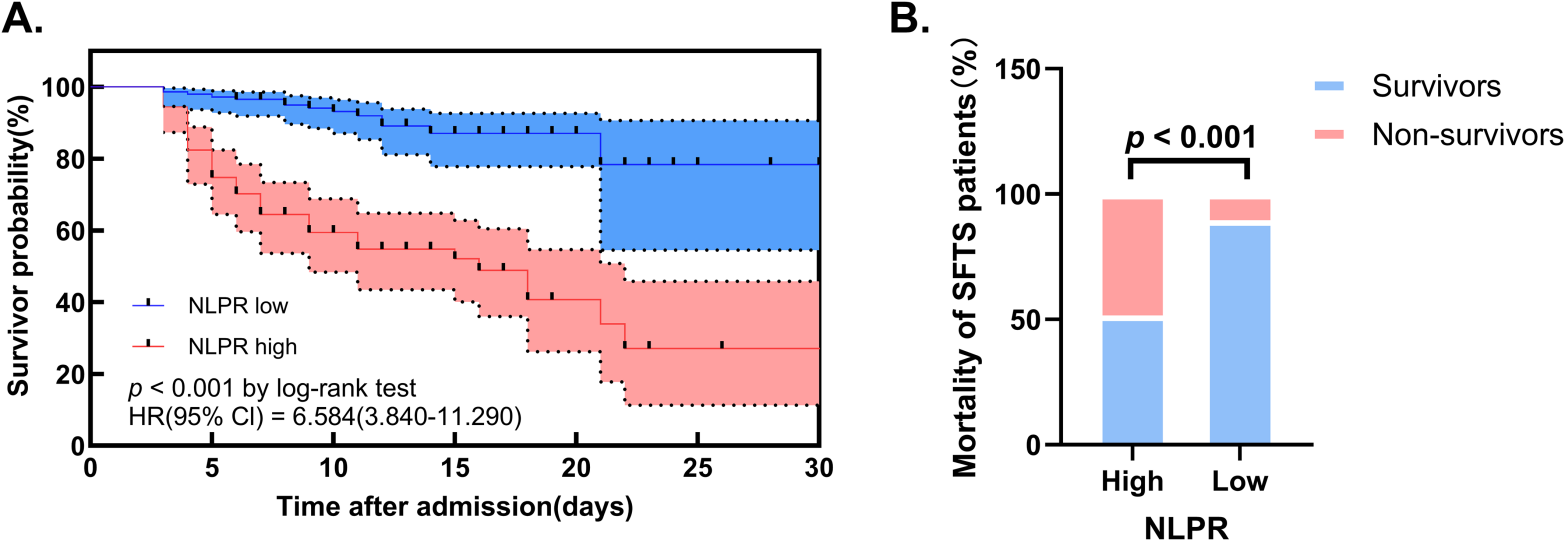
NLPR is associated with poor progress of SFTS. **(A)** Cumulative survival proportion of SFTS patients in high (> 3.76) and low (≤ 3.76) NLPR groups, by days from admission. **(B)** Comparison of 28-day mortality in high and low NLPR patients with SFTS. NLPR, neutrophil-to-lymphocyte-platelet ratio; SFTS, severe fever with thrombocytopenia syndrome; HR, hazard ratios.

Associated factors identified in the longitudinal analysis were further evaluated using ROC curve analysis to assess their predictive performance at each time point. As presented in **Figure 3**, NLPR showed higher AUC values [AUC = 0.802 (95% CI 0.736-0.868)] on day 0 compared with NPAR [AUC = 0.715 (95% CI 0.639-0.791)], Scr [AUC = 0.687 (95% CI 0.612-0.763)], urea [AUC = 0.758 (95% CI 0.687-0.829)], and platelet counts [AUC = 0.696 (95% CI 0.618-0.774)]. NLPR still showed a higher AUC value when compared with the AUC values of the other markers on days 3, 7, and 10, respectively. Additionally, 3.76 was identified as the optimal cutoff at admission, with a sensitivity of 0.754 and specificity of 0.741. Kaplan-Meier survival analysis showed that high NLPR (> 3.76) were associated with poor prognosis among patients with SFTS (HR 6.584 [95% CI 3.840-11.290], *p* < 0.001 by log-rank test) (**Figure 4A**). Moreover, the mortality rate was nearly fourfold higher in the high NLPR group (48.4%) than in the low NLPR group (10.4%) (*p* < 0.001) (**Figure 4B**).

### 3.7 Correlation analysis of NLPR and other laboratory parameters

**Figure 5:**
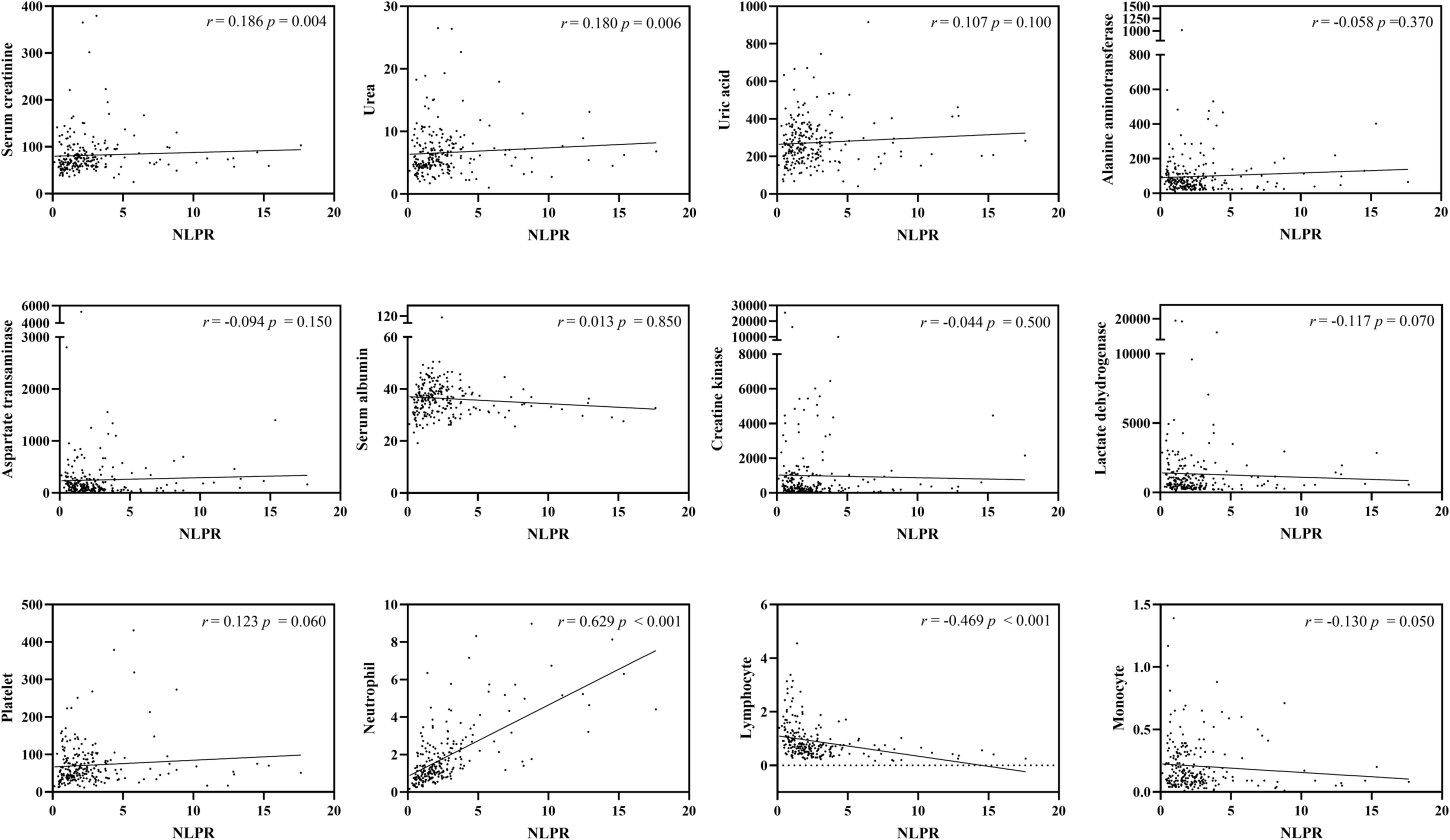
Correlation analysis between the neutrophil-to-lymphocyte-platelet ratio (NLPR) and laboratory parameters.

Variables with *p* < 0.05 in the univariate analysis were further included in the correlation analysis with NLPR. The results showed that NLPR was positively correlated with neutrophils (*r* = 0.629, *p* < 0.001), Scr (*r* = 0.186, *p* = 0.004), and urea (*r* = 0.180, *p* = 0.006) and negatively correlated with lymphocytes (*r* = −0.469, *p* < 0.001) and monocytes (*r* = −0.129, *p* = 0.050) (**Figure 5**).

### 3.8 Efficacy of treatments based on NLPR-stratified patients with SFTS

We further assessed the association between routine treatments and mortality rates in both subgroups of patients with high and low NLPR, after stratification using the optimal NLPR cutoff value. As shown in **Table 5**, in the high-NLPR (> 3.76) subgroup, albumin treatment was associated with a lower risk for mortality after adjustment for confounders (adjusted HR = 0.423 [95% CI 0.195-0.919], *p* = 0.030). However, no significant association between the treatments and mortality was observed in the low NLPR (≤ 3.76) subgroup.

**Table 5:** Cox regression analysis of treatments in patients with NLPR >3.76 or ≤ 3.76.

	Low-NLPR (≤ 3.76, n = 144)		High-NLPR (>3.76, n = 91)	
Treatments	Adjusted HR (95% CI) ^*^	*p* Value	Adjusted HR (95% CI) ^*^	*p* Value
**Ribavirin**	Yes (n = 77)		Yes (n = 45)	
Yes	1.933 (0.567-6.591)	0.292	1.071 (0.577-1.990)	0.828
No				
**Albumin**	Yes (n = 28)		Yes (n = 24)	
Yes	1.311 (0.384-4.479)	0.666	0.423 (0.195-0.919)	0.030
No				
**Platelet**	Yes (n = 90)		Yes (n = 67)	
Yes	3.426 (0.611-19.217)	0.162	0.822 (0.404-1.673)	0.589
No				
**Intravenous immunoglobulin**	Yes (n = 98)		Yes (n = 77)	
Yes	4.267 (0.545-33.392)	0.167	2.514 (0.832-7.597)	0.102
No				
**Corticosteroids**	Yes (n = 20)		Yes (n = 14)	
Yes	0.946 (0.252-3.559)	0.935	0.641 (0.237-1.730)	0.380
No				

* Adjusted by age, sex, history of drinking, history of smoking, cardiovascular and cerebrovascular diseases, and diabetes. SFTS, severe fever with thrombocytopenia syndrome; NLPR, neutrophil-to-lymphocyte-platelet ratio.

## 4 Discussion

SFTS is a rapidly progressive infectious disease with high mortality. In this longitudinal study, laboratory parameters were observed to be changed rapidly in patients with SFTS, and NLPR was identified as a consistently informative predictor of mortality throughout the disease course. Furthermore, receiving albumin treatment was associated with a lower risk for mortality in the high-NLPR subgroup.

To further clarify the prognostic significance of these dynamic changes, we evaluated temporal changes in laboratory parameters and systemic inflammatory indices at predefined clinical time points. The time-points stepwise Cox regression analysis identified Scr, urea, platelets, NLPR, and NPAR as independent associated factors for 28-day mortality. ROC analyses further showed that NLPR consistently demonstrated higher AUC values across the evaluated time points. Mortality in the high-NLPR group was nearly fourfold higher than that in the low-NLPR group after stratification. Therefore, these findings suggest that NLPR may serve as an important marker of prognosis in patients with SFTS.

Laboratory findings on admission suggest marked hematological and biochemical abnormalities in many patients with SFTS. Thrombocytopenia is one of the most prominent features of SFTS. Fang et al. (2023) reported that viral replication within platelets and enhanced platelet activation may contribute to accelerated platelet depletion. Lymphopenia in SFTS may reflect broader adaptive immune impairment and disordered humoral immune responses (Yang et al., 2022a). Additionally, we observed an upward trend in lymphocyte and monocyte counts in the early stages of disease. The trend may represent a compensatory response to infection, as previously reported in patients with bacterial sepsis (Liu et al., 2021) and viral infection (Wong et al., 2021). Previous studies have suggested that neutrophils and lymphocytes play key roles in the inflammatory response, and platelets may further amplify inflammation by interacting with neutrophils (Umut et al., 2023). Platelets may also be closely related to disease progression and clinical outcomes. In trauma patients, platelets have been found to recognize pathogens, recruit leukocytes, release inflammatory mediators, and participate in tissue repair (Chae et al., 2021). Tian et al. reported that failure of the platelet counts to recover to 50×10^9^/L within 5 days was an independent risk factor for death in patients with SFTS (Tian et al., 2025). In our study, temporal changes also indicated a significant recovery of platelet count in the survival group during the late stage.

Previous pathological studies have shown that the DBV antigen is most abundant in the spleen, followed by the kidneys, suggesting that the kidney is an important target organ for DBV (Li et al., 2018). SFTS can cause varying degrees of renal parenchymal injury and may progress to acute kidney injury in severe cases (Xu et al., 2018). Notably, albumin levels of the non-survivors remained consistently low. Hypoalbuminemia during systemic inflammation may result from increased capillary permeability, poor nutritional status, and impaired hepatic synthetic function (Feng et al., 2022).These laboratory abnormalities indicated multiorgan dysfunction in the non-survivor group. Consistent with this possibility, in ferret models, DBV nucleoproteins and viral RNA have been detected in multiple organs, suggesting that DBV can disseminate widely throughout the body and involve multiple organs (Park et al., 2019).

Albumin therapy may have biological effects beyond nutritional support. Recent studies have suggested that hypoalbuminemia is closely related to systemic inflammation and disease severity rather than nutritional status alone (Allison and Lobo, 2024). In addition to maintaining plasma oncotic pressure, albumin may stabilize endothelial function and microcirculation, preserve endothelial glycocalyx integrity, and exert antioxidant and anti-inflammatory properties (Aldecoa et al., 2020). Because NLPR may reflect systemic inflammatory burden, these biological properties may provide a plausible context for the observed association between albumin therapy and a lower risk of mortality in the high-NLPR subgroup. However, this finding should be interpreted cautiously because patients receiving albumin therapy may have differed from those not receiving albumin in terms of disease severity, supportive care, nutritional status, organ dysfunction, or underlying comorbidities, which may have influenced the observed association. Therefore, this result should be considered hypothesis-generating rather than evidence of a direct therapeutic effect.

Laboratory findings may change rapidly after admission, which may partly explain the limitations of cross-sectional studies. Non-survivors often exhibit more pronounced laboratory abnormalities during the acute phase, which may explain the substantial heterogeneity and fluctuations observed in clinical parameters (Chinese Center for Disease Control and Prevention, 2024). In our study, renal function markers showed the most pronounced abnormalities on day 7, whereas liver and cardiac enzyme levels peaked earlier. He et al. (2021) reported that renal–related indicators increased from disease onset and peaked by day 10. Li et al. (2014) found that peaks in liver and cardiac enzyme levels in non-survivors occurred at admission. These findings suggest that liver and cardiac injury may occur earlier during the acute phase of SFTS, whereas renal dysfunction may become more prominent as the disease progresses. Interestingly, NLPR and PNR showed greater fluctuations during the disease course, especially on day 7, which may have facilitated better patient stratification.

For systemic inflammation indices, NLPR may capture the core pathophysiological features of SFTS more comprehensively by integrating neutrophils, lymphocytes, and platelets may better reflect the combined effects of inflammation, immune dysregulation, and thrombocytopenia, which are central features of SFTS pathophysiology. Shi et al. (2022) applied NLPR in sepsis cohorts and demonstrated its utility for mortality prediction, supporting the value of dynamic monitoring. In addition, NLR has received significant attention in previous studies and is associated with short-term mortality risk in patients with SFTS (Liu et al., 2022). In contrast, although several inflammatory indices were assessed in a study by Yang et al. (2022b), which is a small cohort enrolled 82 patients, no significant differences were observed between the two groups, this discrepancy may be partly related to the limited sample size and the use of few time points, which may be insufficient to capture the rapid and dynamic changes in inflammatory indices during SFTS progression. In another comparative analysis, the prognostic performance of PLR and SII was relatively limited (Guo et al., 2025). Owing to limitations in sample size and the number of included indicators, data on the application of systemic inflammatory indices in the prognosis of SFTS remain insufficient. Moreover, previous prognostic models for SFTS, such as the mortality prediction score developed by Chen et al. (2025), incorporated multiple clinical and laboratory variables. Compared with cross-sectional study design and complexity of scoring models, our study emphasizes the longitudinal evidence by evaluating the dynamic prognostic value of routinely hematology-derived systemic inflammatory indices. This approach highlights the potential clinical utility of NLPR as a simple and dynamically assessable prognostic index during hospitalization. Further studies with direct comparative analyses are needed to determine how NLPR performs relative to established prognostic models in different clinical settings.

This study had several limitations. First, the absence of external validation may limit the generalizability of our findings. Second, the single-center design may have introduced selection bias. Third, viral load data were not included because of limited data availability.

To the best of our knowledge, this is the first longitudinal study to evaluate the prognostic value of systemic inflammatory indices for mortality of SFTS. A significant finding of this study was that NLPR was a consistently informative predictor of mortality throughout the disease course. The secondary finding was that albumin therapy was associated with a lower risk of death in the high-NLPR subgroup. The utility of NLPR as a stage-stratified prognostic index and its potential role in informing future studies on albumin therapy warrant further investigation in SFTS.

## Data Availability

The original contributions presented in the study are included in the article/**Supplementary Material**. Further inquiries can be directed to the corresponding author.
