# Supplementary material for: NLPR as a predictor of poor prognosis in patients with severe fever with thrombocytopenia syndrome: a prospective longitudinal study
Source: Front Cell Infect Microbiol. 2026 Jun 12;16:1848735. doi: 10.3389/fcimb.2026.1848735 (PMC13303888; doi:10.3389/fcimb.2026.1848735)
Supplement: Supplementary file 1 [file Table1.docx]

Supplementary Table

**Table S1. Comparisons of non-specific symptoms in severe fever with thrombocytopenia syndrome patients.**

| Symptoms, n (%) | Total (n=235) | Survivors (n=174) | Non-survivors (n=61) | *p* Value |
| --- | --- | --- | --- | --- |
| Headache | 38 (16.2) | 29 (16.7) | 9 (14.8) | 0.727 |
| Cough | 39 (16.6) | 26 (14.9) | 13 (21.3) | 0.250 |
| Skin changes | 38 (16.2) | 22 (12.6) | 16 (26.2) | 0.013 |
| Sore throat/Swelling | 5 (2.1) | 3 (1.7) | 2 (3.3) | 0.607 |
| Chills and rigors | 99 (42.1) | 72 (41.4) | 27 (44.3) | 0.695 |
| Fatigue | 180 (76.6) | 131 (75.3) | 49 (80.3) | 0.424 |
| Abdominal pain and diarrhea | 116 (49.4) | 81 (46.6) | 35 (57.4) | 0.146 |
| Nausea/Vomiting | 89 (37.9) | 65 (37.4) | 24 (39.3) | 0.783 |
| Gingival bleeding | 17 (7.2) | 10 (5.7) | 7 (11.5) | 0.137 |
| Muscle soreness | 73 (31.1) | 51 (29.3) | 22 (36.1) | 0.327 |
| Neurological symptoms | 36 (15.3) | 11 (6.3) | 25 (41.0) | < 0.001 |
| Chest tightness | 24 (10.2) | 10 (5.7) | 14 (23.0) | < 0.001 |
| Palpitations | 16 (6.8) | 11 (6.3) | 5 (8.2) | 0.838 |
| Lymphadenopathy | 46 (19.6) | 32 (18.4) | 14 (23.0) | 0.440 |
